# Behavioural responses to heat in an arid-zone bird vary with thermal, hygric and social factors

**DOI:** 10.1007/s00442-026-05888-4

**Published:** 2026-04-06

**Authors:** J. R. D. Whyte, A. E. McKechnie, J. K. Crossley, S. J. Cunningham

**Affiliations:** 1 FitzPatrick Institute of African Ornithology, DSI-NRF Centre of Excellence, University of Cape Town, Cape Town, South Africa; 2 DSI-NRF Centre of Excellence at the FitzPatrick Institute, Department of Zoology and Entomology, University of Pretoria, Pretoria, South Africa; 3 South African Research Chair in Conservation Physiology, South African National Biodiversity Institute, Pretoria, South Africa

**Keywords:** *Plocepasser mahali*, White-browed sparrow-weaver, Climate change, Microhabitat, Thermoregulation

## Abstract

The severity of fitness costs resulting from behavioural trade-offs between heat dissipation and activities such as foraging is increasing with advancing climate change. In terrestrial habitats, shade and water may buffer individuals from the negative effects of heat exposure, as may load-lightening in group-living species. We tested the hypothesis that thermal and hygric properties of home ranges (shade and water availability) and social factors (group size) influence the costs associated with hot weather, using a population of white-browed sparrow-weavers (*Plocepasser mahali*) in the southern Kalahari Desert. Across all sparrow-weaver groups, heat avoidance (shade-seeking) and dissipation (panting) behaviours increased with increasing air temperature (*T*_air_), whereas foraging declined. Birds occupying shadier home ranges delayed the onset of panting to higher *T*_air_ and foraged less while maintaining overall peck rates. Birds with access to water foraged more, maintained higher peck rates and sought shade at higher *T*_air_ compared to birds without. However, they did not pant more, making the mechanism underpinning their increased foraging effort unclear. Birds in larger groups both panted more overall and sought shade at lower *T*_air_ than birds in smaller groups but maintained similar overall peck rates. Taken together, these results suggest birds in shadier home ranges can forage more efficiently, buffering foraging costs at high *T*_air_. Our data therefore suggest that some impacts of increasing *T*_air_ under climate change can be buffered by shade availability, but the impacts of water availability and social factors are less clear.

## Introduction

Small diurnal endotherms occupying hot environments rely on behavioural thermoregulation to reduce external and internal heat loads when environmental temperatures approach or exceed their normothermic body temperatures (*T*_b_) (Dawson and Schmidt-Nielsen 1964; Dawson and Bartholomew 1968; Fisher et al. 1972). Behavioural responses to heat exposure may reduce heat gain (e.g. shade-seeking and curtailing activities such as foraging and social interactions (Davies 1982; Carroll et al. 2015; Smit et al. 2016) or increase heat dissipation (e.g. panting, wing-drooping (Bartholomew et al. 1968; Richards 1970; Pattinson et al. 2020). These heat dissipation behaviours permit birds to defend *T*_b_ at sublethal levels during heat exposure but often entail opportunity costs through behavioural trade-offs with foraging (e.g. du Plessis et al. 2012; Wiley and Ridley 2016; Cunningham et al. 2021). Foraging opportunity costs associated with heat waves result in progressive loss of body mass in adults and/or reduced breeding success associated with reduced nestling provisioning and consequent decreases in nestling survival, growth rates and fledgling quality (van de Ven et al. 2019, 2020a, b; Bourne et al. 2020a; Sharpe et al. 2021). With advancing climate change, increases in heat-associated fitness costs are expected to drive declines in desert bird communities (Conradie et al. 2019).

Although the opportunity costs associated with hot periods have been quantified for species on several continents (e.g. Bozinovic et al. 2000; du Plessis et al. 2012; Edwards et al. 2015; Pigeon et al. 2016), it remains less clear how these costs vary intraspecifically. The spatial structure of thermal landscapes and the availability of cool microsites may buffer individuals from the effects of high temperatures under current and future climates (Sears 2005; Sinervo et al. 2010; Carroll et al. 2015, 2016; Sears and Angilletta 2015). In spatially heterogeneous thermal landscapes, home ranges occupied by conspecifics vary in their thermal properties on account of factors such as vegetation cover (Sears 2005; Carroll et al. 2015; Sears and Angilletta 2015). The dynamics of behavioural trade-offs between foraging and heat dissipation may vary with shade availability and, a priori, these trade-offs may be expected to be less severe for individuals occupying better-shaded territories where they can more readily retreat to cool microsites between foraging bouts.

In addition to vegetation cover and the associated shade, another variable likely to cause intraspecific variation in thermoregulatory costs across territories is water availability. As small, diurnal endotherms, birds in hot environments face substantial evaporative cooling requirements (Dawson and Schmidt-Nielsen 1964; Wolf and Walsberg 1996) and trade off dehydration avoidance (minimising water losses) against hyperthermia avoidance (evaporative cooling to maintain *T*_b_ at sublethal levels). Increasing cooling costs are thought to have driven declines in a desert bird community over the last century (Riddell et al. 2019) and are predicted to expose many arid-zone species to greatly increased risks of lethal dehydration in future (Albright et al. 2017; Conradie et al. 2020). During hot weather, the challenges associated with maintaining water balance might be less severe for individuals whose home ranges include surface water sources such as waterholes or livestock troughs and which can replenish body water by drinking (Fisher et al. 1972; Abdu et al. 2018).

Intraspecific variation in thermoregulatory costs may also interact with variables related to sociality. Bet hedging and load-lightening in group-living cooperative species have been proposed to buffer costs associated with harsh environmental conditions and high temperatures in arid habitats (Covas et al. 2008; Rubenstein 2011; Clutton-Brock and Manser 2016). For example, sharing of vigilance behaviour between group members could allow individuals in larger groups to maintain higher foraging efficiency or intensity than those in smaller groups, reducing the time they need to spend foraging and increasing their capacity to avoid high heat loads by reducing activity and seeking shade during hot periods. However, the benefits of larger group size in some years may be negated by costs in others when intragroup competition for food is more severe (Rubenstein and Lovette 2007; Rubenstein 2011; Riehl and Smart 2022, but see also Bourne et al. 2020b).

Understanding how the fitness costs of hot weather among individuals vary with abiotic and biotic factors is essential for predicting species’ persistence in hot environments. We hypothesised that thermal and hygric properties of home ranges, as well as group size, influence behavioural trade-offs during hot weather. We tested this hypothesis using white-browed sparrow-weavers (*Plocepasser mahali*; hereafter, sparrow-weavers) in the Kalahari Desert of southern Africa. Sparrow-weavers are cooperatively breeding birds with variable group sizes (du Plessis 2005) and are opportunistic drinkers that use surface water if available within their home ranges (Smit et al. 2013, 2019). Specifically, we tested the following predictions: (1) the slopes of declines in foraging activity and increases in heat dissipation behaviours as functions of air temperature are shallower for birds in better-shaded home ranges; (2) birds with water available in their home ranges forage more and seek shade less during hot periods compared to birds without access to water, facilitated by higher rates of panting (evaporative cooling); (3) larger group size benefits individuals by increasing foraging efficiency and thus buffering foraging-thermoregulation trade-offs.

## Methods

### Study site and study species

We studied sparrow-weavers at the 75 km^2^ Murray Guest Farm near the town of Askham in the southern Kalahari, South Africa (26°59′ S, 21°51′ E, elevation ~ 870 m) during the austral summer (October to March) of 2021/22. The site is characterised by arid savanna vegetation with scattered trees, including *Vachellia erioloba, V. haematoxylon, Boscia albitrunca*, and shrubs, including *Senegalia mellifera* and *Rhigozum trichotomum*, growing on grassy red sand dunes. The region experiences mean daily maximum summer air temperatures (*T*_max_) of 34.9 ± SD 1.19 °C (for the period 1991–2020, Pattinson et al. 2022), with mean annual rainfall of 213.9 ± SD 102.0 mm (1993–2020, Pattinson et al. 2022), falling mostly during the summer. A Davis VantagePro2 weather station was placed at the site to record air temperature (*T*_air_; °C) at 10-min intervals throughout the study period.

Sparrow-weavers are medium-sized (~ 40 g in the Kalahari, Smit et al. 2013) cooperatively breeding passerines that forage terrestrially on seeds and small insects. Cooperative groups consist of a single dominant breeding pair and up to 12 helpers (Collias and Collias 1978; Ferguson and Siegfried 1989; du Plessis 2005). Groups build a number of distinctive tubular “croissant-shaped” domed nests from dried grass in a central tree (or adjacent trees) within the home range, and individuals rarely venture more than a few hundred metres from their nest tree (du Plessis 2005). Nests with two entrances are used for roosting year-round (one bird per nest). This distinguishes them from breeding nests, which are sealed at one end when the dominant female is ready to lay eggs (Ferguson and Siegfried 1989).

We studied 15 groups of sparrow-weavers, ranging in size from 2 to 10 birds. Across these groups’ home ranges, there was variation in density of trees, contributing to different shade profiles. Five of the 15 groups had water available within 200 m of their nest tree in livestock watering troughs, whereas 10 did not. Birds (*n* = 64, body mass = 41.1 ± 3.3 g) were caught directly from their roost nests at night and colour-ringed for individual identification with one uniquely numbered aluminium and three plastic colour rings in a unique combination. After ringing, birds were returned immediately into their roost nests where they would typically remain until morning.

### Behavioural observations

Individually colour-ringed birds were habituated to the close presence of humans (~ 5 m), following Ridley and Raihani (2007). We then conducted instantaneous scan samples (hereafter ‘scans’) and focal observations (hereafter ‘focals’) of the ringed birds following Altmann (1974) to investigate relationships between *T*_air_, foraging and thermoregulatory behaviours. Data were collected from 1 to 2 groups per day, ensuring the same group was not visited on consecutive days. We randomised the order in which groups were visited at the beginning of the season but ensured all groups were visited across a broad range of daily maximum *T*_air_ (*T*_max_) as the season progressed (i.e. creating a stratified random schedule based on *T*_max_). During focals and scans, the order of observations of individuals was randomised by leg band number.

Focals (*n* = 539 from 64 birds in 15 groups) were carried out during three periods (“Focal Blocks”) each day (5:30–8:30, 12:30–15:30 and 16:30–19:30). Each focal involved continuous recording of a single bird’s behaviour for 15 min (mean ± SD = 15.32 ± 0.06 min). During focals, time allocated to foraging versus other mutually exclusive behaviours was recorded using a digital voice recorder and then transcribed with a smartphone into Cybertracker software (CyberTracker Conservation, 2021) following van den Ven et al. (2019). We also recorded time spent panting (a behaviour facilitating evaporative cooling, involving gaping during breathing, Smit et al. 2016), the bird’s height above ground to the nearest 1 m, and whether the bird was in the sun or shade. We counted the number of peck or probe actions made by birds during foraging, while in sun or shade and panting or not. These were used to quantify the peck rate of foraging individuals as a proxy for foraging intensity, and to quantify the number of pecks performed per focal observation as a proxy for foraging efficiency. The prey items and seeds ingested by the birds were usually too small to accurately record, so true foraging efficiency in terms of biomass or number of food items eaten per unit time could not be estimated. If the bird moved out of sight for > 2 min, the focal was terminated.

Scans (*n* = 2280 from 64 birds in 15 groups) were conducted for each visible bird in the focal group at the start and end of each focal block and between each 15 min focal. Scans involved taking an instantaneous observation of behaviour at the moment of the scan, in discrete categories (including foraging, flying, preening, etc.), plus presence or absence of panting, sun/shade exposure and height off the ground. The location (within 5 m of the bird) for each scan sample was recorded using a handheld GPS unit.

### Home-range surveys

After 2 weeks of field work, GPS locations extracted from the scan samples (*n* = 313) were plotted in QGIS (version 3.16.8-Hannover, 2021), confirming that the birds generally remained within 200 m of their nest tree (as also indicated by Plessis 2005). In QGIS, we therefore created circular “home range buffers” of 200 m radius around the nest tree for each group. We overlaid a 30 × 30 m grid within each buffer and extracted GPS locations for each grid point (*n* = 157 per group). We then visited each grid point in the field and recorded perch availability in 50 cm height increments from the ground (height = 0 cm) to the highest available perch. The proportion of these perches shaded at midday by plants or other structures overhead determined the “midday shade” index for each group. We also recorded whether surface water was available within 200 m of each nest tree (e.g. livestock watering troughs).

### Statistical analyses

All statistical analyses were carried out in the R statistical environment (R Core Team, 2022) using the RStudio interface (RStudio Team, 2020), core packages and the packages *ggplot2* (Wickham 2016), *lme4* (Bates et al. 2015), *car* (Fox and Weisberg 2019) and *interactions* (Long 2019). We assessed correlations between predictor variables and did not fit correlated variables within the same model. We also checked model variance inflation factors after fitting (all were below 2.5). All continuous predictors were scaled. We verified model assumptions were met by checking for overdispersion and normality of residuals. P-values < 0.05 were taken as statistically significant and data are presented as mean estimates ± 1 standard error (SE), unless otherwise stated.

### Scan data

General linear mixed models (GLMMs) with binomial distribution and logit link were fitted to the scan data. Focal Block and *T*_air_ (i.e. *T*_air_ recorded by the onsite weather station at the time of the scan, to the nearest 10 min) predictor variables were correlated, with morning *T*_air_ cooler than the rest of the day. Akaike Information Criterion (AIC) values revealed that models with *T*_air_ as a predictor variable substantially outperformed models with Focal Block for foraging and panting behaviours (> 14 Δ AIC and > 98 Δ AIC, respectively), while for shade-seeking models, Focal Block Δ AIC was lower than *T*_air_ Δ AIC. For consistency, we used *T*_air_ in all models instead of Focal Block as it was (a) our core variable of interest, (b) the overall better explanatory variable, and (c) because all three focal blocks were evenly represented in the scan sample data set (*n* = 732 observations from the morning focal block, *n* = 779 from midday and *n* = 769 from afternoon), ensuring no focal block was overrepresented in the sample.

Models were fitted for each of the response variables: foraging (0 = not foraging, 1 = foraging), shade-seeking (0 = in sun, 1 = in shade) and panting (0 = not panting, 1 = panting); including (as predictors) the interactions between *T*_air_ and midday shade availability; *T*_air_ and water (0 = no water available, 1 = water available); and *T*_air_ and group size (number of birds in the focal individual’s group). Dominance (0 = subordinate, 1 = member of the dominant breeding pair) and sex (M, F) were also included to control for possible effects of these on the response variable, as both have been shown to correlate with thermoregulatory behaviour in other species (Alonso et al. 2016; Cunningham et al. 2017). Random terms (bird ID nested within group ID; and date) were included to account for repeated sampling. If random terms caused singular fit errors, the random effects structure was simplified until the model could be run successfully, following Bolker (2015). This ultimately meant models for foraging were fitted with random terms bird ID nested within group ID and date; shade-seeking models with random terms bird ID nested within group ID; and panting models with random term bird ID only. We assessed the impact of simplifying the random error structure for foraging and shade-seeking models and found no qualitative differences in the results when models were fitted with the random term bird ID alone, so we present the most complex/complete models.

After fitting the models, non-significant interactions were removed to allow interpretation of main effects. Interactions between variables were assessed visually using the R package *interactions* (Long 2019). Sample sizes for scan data analyses were *n* = 2280 scan observations of 64 individual birds across 15 groups.

### Focal data

GLMMs with negative binomial error distribution were fitted to peck rate data to assess the number of pecks performed by individual sparrow-weavers per focal observation. A model was fitted including interactions between (a) *T*_air_ and midday shade availability, (b) *T*_air_ and water availability, and (c) *T*_air_ and group size. The model also included dominance and sex, and the random term bird ID (additional random terms Group ID and Date caused singular fit errors). Non-significant interactions were subsequently removed, to allow interpretation of main effects. Sample size for this analysis was 538 focals from 64 birds in 15 groups.

We used paired t-tests to compare (1) peck rates per unit time foraging in sun versus shade, where birds foraged in both exposure categories within the same focal (*n* = 171 paired observations of 56 individuals); and (2) peck rates per unit time foraging for birds panting versus not panting, where both behaviours had been observed in the same focal (*n* = 13 paired observations of 11 individuals). Data were paired by focal to control for variables such as bird and group identity, weather conditions, and background food availability which are likely to remain constant within the 15 min focal periods.

## Results

### Habitat features and weather conditions

The average home range size of the 15 sparrow-weaver groups was 0.04 ± 0.03 km^2^. Home-range polygons drawn using the full scan sample bird location dataset at the end of the study (*n* = 2280 locations) showed 97.3 ± 6.4% overlap with the 200-m radius home range buffers set out at the beginning. The percentage of perches shaded at midday ranged from 1.9% to 19.7% across home ranges (mean = 11.8 ± 7.2%). Five groups (comprising 39.4% of individuals in the study) had permanent access to surface water in the form of livestock watering troughs within their home ranges. Maximum daily *T*_air_ ranged from 21.8 to 40.4 °C during the study, but humidity remained low throughout (mean dew point 8.7 ± 7.5 °C).

### Foraging behaviour

**Fig. 1:**
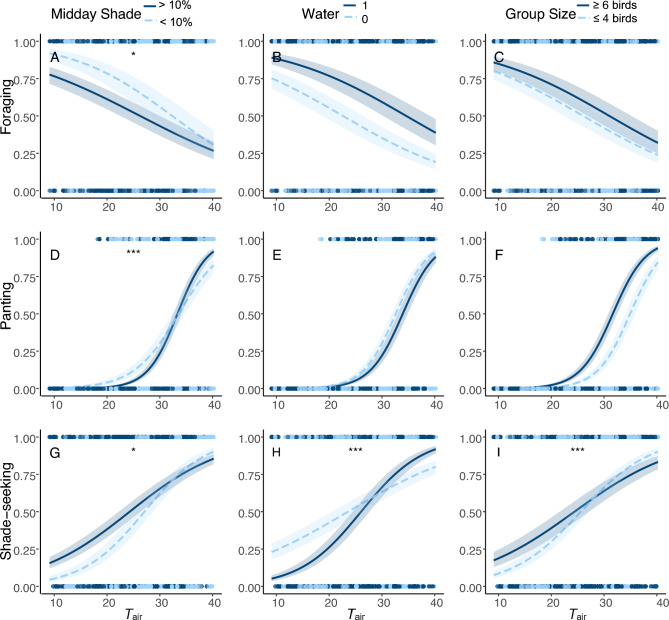
Relationships between foraging, panting and shade-seeking behaviours of White-browed Sparrow-weavers *Plocepasser mahali,* and air temperature (*T*_air_ °C), habitat (shade and water availability; 1 = water available, 0 = no water available) and social factors. Asterisks indicate significant interactions between *T*_air_ and the variable in the column heading (* *p* < 0.05, *** *p* < 0.001). The continuous variable ‘midday shade’ is split into low (< 10%) and high (> 10%) and ‘group size’ into small (≤ 4 birds) and large (≥ 6 birds) categories for ease of visualisation, but significance values are drawn from models fitted to continuous data for both variables. *n* = 2280 scan samples of 64 adult sparrow-weavers from 15 groups

Increasing *T*_air_ was correlated with a reduction in the probability that sparrow-weavers were observed foraging, but this relationship was modified by shade availability. Birds in shadier home ranges experienced a shallower decline in the probability of foraging as *T*_air_ increased and were significantly less likely to forage at cooler *T*_air_ than birds in more sun-exposed home ranges (Table 1, Fig. 1A). Sparrow-weavers with access to surface water within 200 m of their nest tree were significantly more likely to forage at any time compared to those without water (Fig. 1B), but there was no interaction between water availability and *T*_air_. Sex, dominance status and group size did not influence the likelihood of foraging (Table 1).

**Table 1:** Factors correlated with the probability of foraging by adult White-browed Sparrow-weavers, *Plocepasser mahali*

Variable	Estimate	Std error	Z value	*p* value
Intercept	−0.320	0.249	−1.286	0.199
*T*_air_	**−0.669**	**0.056**	**11.965**	** < 0.001**
Midday shade	−0.172	0.160	1.076	0.282
Water	**0.983**	**0.368**	**2.674**	**0.008**
Group size	0.227	0.170	1.333	0.183
Sex	−0.026	0.113	0.229	0.820
Dominance	−0.146	0.120	1.221	0.222
*T*_air_: midday shade	**0.136**	**0.055**	**2.479**	**0.013**

Water = 0 (none), sex = F and dominance = 0 (subordinate) are set as baselines and significant relationships are indicated with bold text. Results are based on general linear mixed models with binomial distribution and logit link. The global model included interactions between: air temperature (*T*_air_) and midday shade; *T*_air_ and water; and *T*_air_ and group size. Non-significant interactions were removed

*n* = 2280 scan samples on 64 adult sparrow-weavers from 15 groups

### Panting behaviour

There was a positive correlation between *T*_air_ and the probability that sparrow-weavers were observed panting during scans. This relationship was modified by the availability of shade, such that birds in shadier home ranges began panting at higher *T*_air_ than those in sunnier home ranges (a statistically significant interaction; Table 2, Fig. 1D). Sparrow-weavers from larger groups were significantly more likely to be observed panting than those from smaller groups (Fig. 1F), but there was no interaction between group size and *T*_air_. The likelihood of panting was not related to sex, dominance status or (surprisingly) surface water availability (Table 2).

**Table 2:** Factors correlated with the probability of panting by adult White-browed Sparrow-weavers, *Plocepasser mahali*

Variable	Estimate	Std error	Z value	*p* value
Intercept	**−1.779**	**0.248**	**7.171**	** < 0.001**
*T*_air_	**2.620**	**0.138**	**18.928**	** < 0.001**
Midday shade	**−0.281**	**0.139**	**2.021**	**0.043**
Water	−0.424	0.285	1.488	0.137
Group size	**0.594**	**0.150**	**3.965**	** < 0.001**
Sex	−0.108	0.246	0.438	0.662
Dominance	0.199	0.253	0.786	0.432
*T*_air_: Midday shade	**0.552**	**0.117**	**4.711**	** < 0.001**

Water = 0 (none), sex = F and dominance = 0 (subordinate) are set as baselines, significant relationships are indicated with bold text. Results are based on general linear mixed models with binomial distribution and logit link. The global model included interactions between: air temperature (*T*_air_) and midday shade; *T*_air_ and water; and *T*_air_ and group size. Non-significant interactions were removed

*n* = 2280 scan samples on 64 adult sparrow-weavers from 15 groups

### Shade-seeking behaviour

The probability of shade-seeking was described by interactions between water and *T*_air_, midday shade and *T*_air_, and group size and *T*_air._ The interaction between water and *T*_air_ had a significant positive correlation with shade-seeking (Table 3; Fig. 1H), while the interactions between *T*_air_ and group size and *T*_air_ and midday shade had significant negative correlations with shade-seeking (Table 3, Fig. 1G, I). Birds in groups with no access to water were much more likely to be observed in the shade at cooler *T*_air_ than those with access to water, while individuals from larger groups sought shade at higher *T*_air_ than those in smaller groups. Birds in shadier home ranges were more likely to be seen in the shade at lower *T*_air_ than those in sunnier home ranges, though this difference disappeared at the hottest air *T*_air_ (Table 3, Fig. 1G).

**Table 3:** Factors correlated with the probability of shade-seeking by adult White-browed Sparrow-weavers, *Plocepasser mahali*

Variable	Estimate	Std error	z value	*p* value
Intercept	0.237	0.197	1.201	0.230
*T*_air_	**0.682**	**0.072**	**9.464**	** < 0.001**
Midday shade	0.176	0.121	1.451	0.147
Water	−0.092	0.283	0.324	0.746
Group size	−0.137	0.146	0.940	0.347
Sex	0.015	0.132	0.111	0.912
Dominance	0.172	0.137	1.251	0.211
*T*_air_: midday shade	**−0.130**	**0.054**	**2.417**	**0.016**
*T*_air_: water	**0.716**	**0.124**	**5.760**	** < 0.001**
*T*_air_: group size	**−0.241**	**0.062**	**3.882**	** < 0.001**

Water = 0 (none), sex = F and dominance = 0 (subordinate) are set as baselines, significant relationships are indicated with bold text. Results are based on a general linear mixed model with binomial distribution and logit link

*n* = 2280 scan samples of 64 adult sparrow−weavers from 15 groups

### Pecks per focal

The number of pecks performed by individual sparrow-weavers per focal observation declined significantly as *T*_air_ increased and birds with access to water performed significantly more pecks per focal than those without. This was consistent with birds with water in their home ranges being significantly more likely to be foraging during scan samples compared to those without water, across all *T*_air_ (Fig. 1B). There were no significant interactions between *T*_air_ and group size, water or shade availability, nor were there main effects of shade availability, group size, dominance, or sex (Table 4).

**Table 4:** Factors correlated with peck rates per focal in adult White-browed Sparrow-weavers, *Plocepasser mahali*

Factor	Estimate	Std error	z value	*p* value
Intercept	**1.562**	**0.134**	**11.623**	** < 0.001**
*T*_air_	**−0.163**	**0.055**	**2.965**	**0.003**
Midday shade	−0.054	0.064	0.837	0.402
Water	**0.701**	**0.152**	**4.595**	** < 0.001**
Group size	0.053	0.079	0.670	0.503
Sex	0.090	0.131	0.688	0.491
Dominance	−0.031	0.136	−0.231	0.817

Water = 0, sex = F and dominance = 0 (subordinate) are set as baselines. General linear mixed model with negative binomial distribution and log link. Results are based on general linear mixed models with binomial distribution and logit link. The global model included interactions between: air temperature (*T*_air_) and midday shade; *T*_air_ and water; and *T*_air_ and group size. Non-significant interactions were removed

*n* = 538 focals on 64 adult sparrow-weavers from 15 groups

### Influence of panting and shade-seeking on foraging intensity

**Fig. 2:**
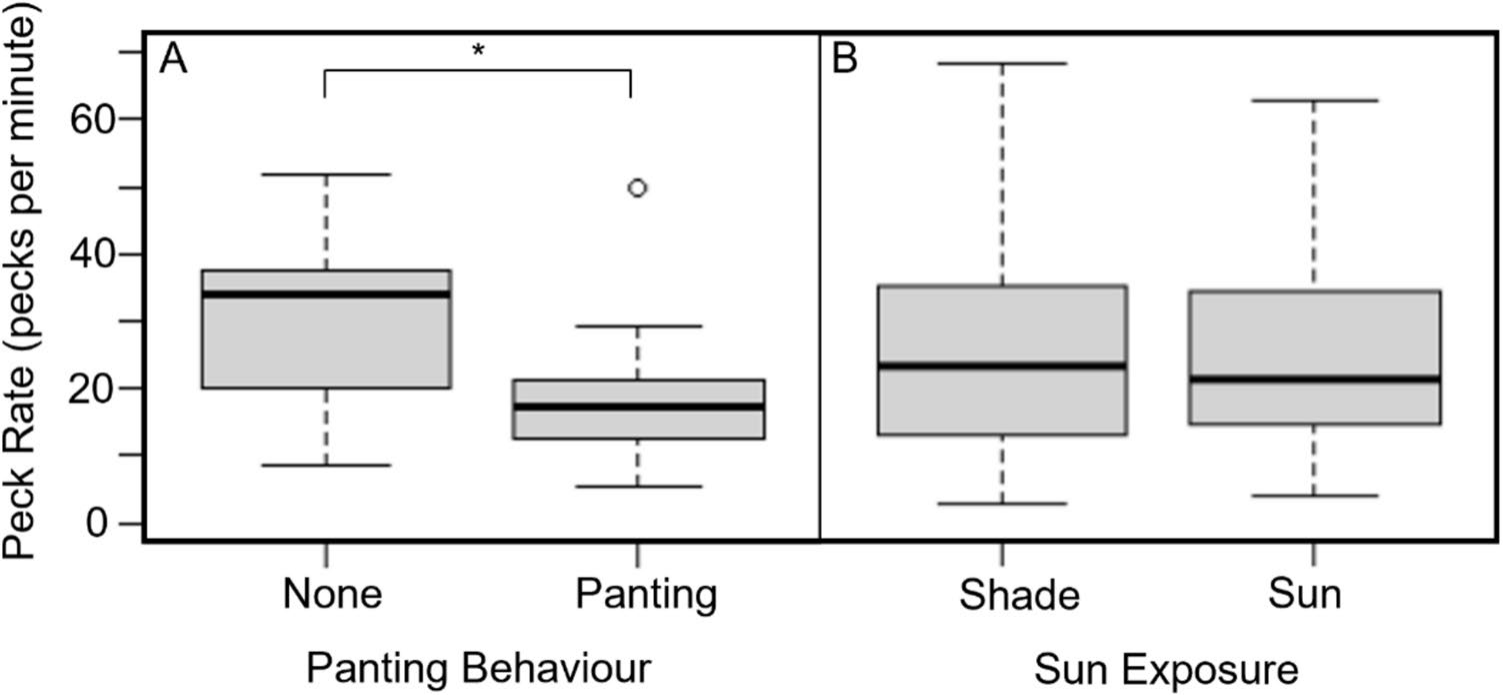
Box plots of foraging intensity (pecks min^−1^ while foraging) in adult White-browed Sparrow-weavers (*Plocepasser mahali*) while (**A**) panting or not panting while foraging and (**B**) foraging in the shade or in the sun. Boxes represent the interquartile range, with the thick black line indicating median peck rate. Whiskers represent maximum and minimum values and the dot in panel A represents an outlier more than 1.5 × the upper quartile. The asterisk in panel A indicates the significant difference between mean peck rates when birds were panting versus not panting, within the same focal observation. Panting data: *n* = 13 paired observations of 11 birds; sun exposure data: *n* = 171 paired observations of 56 birds

Within the same 15 min focals (i.e. under comparable *T*_air_ and foraging conditions) foraging intensity, measured in pecks min^−1^ during foraging, was lower when birds were panting than when they were not (paired t-test, t = 2.12, *p* = 0.04, Fig. 2A). However, there was no significant difference in peck rates during foraging in sunny versus shady locations within the same focal (paired t-test, t = 0.51, *p* = 0.61, Fig. 2B).

## Discussion

The effects of shade and water availability on relationships between *T*_air_, foraging and heat dissipation behaviours among free-ranging sparrow-weavers supported our hypothesis that thermal and hygric properties of home ranges influence behavioural trade-offs during hot weather. The shallower declines in foraging with increasing *T*_air_ among birds occupying shadier home ranges supported our first prediction. The data also supported our second prediction that water availability is associated with increased foraging and decreased shade-seeking during hot periods, but this was apparently not achieved through greater capacity for evaporative cooling. Sparrow-weavers with access to water did not pant more than conspecifics without access. Finally, contrary to our third prediction, there was no evidence that birds in larger groups achieved higher peck rates or foraged less overall, though they panted more overall and sought shade at cooler *T*_air_ than birds in smaller groups. Taken together, our data suggest shade availability buffers trade-offs between thermoregulation and foraging in this species, but the potential for water availability or larger group sizes to do so is less clear.

The reduced foraging intensity (i.e. peck rates) we observed in sparrow-weavers while panting but not when foraging in shade provides further evidence that thermoregulatory behaviours compromise food intake rates (e.g. du Plessis et al. 2012; van de Ven et al. 2019), but also reveals that costs associated with thermoregulatory behaviour may be species- and perhaps context-specific. For example, reductions in foraging intake while panting seem common in ground-foraging birds (e.g. southern pied babblers (*Turdoides bicolor*, du Plessis et al. 2012), southern yellow-billed hornbills (*Tockus leucomelas*, van de Ven et al. 2019), several *Calendulauda* larks (Kemp et al. 2020; Roberts 2021) and white-browed sparrow-weavers, (this study), but appear not to affect foraging rates in species which hawk prey from perches (e.g. fork-tailed drongos, Murphy, Flower and Cunningham *unpubl. data*)—perhaps because panting may interfere less with a sit-and-wait foraging strategy targeting infrequent, high-value prey compared to a strategy requiring near-constant use of the beak. Reductions in foraging intake while shade-seeking have been documented in common fiscals (Cunningham et al. 2015), rufous-eared warblers (*Malcorus pectoralis* Pattinson and Smit 2017)*,* and southern yellow-billed hornbills (van de Ven et al. 2019) and may correlate with reduced body condition (van de Ven et al. 2019). Changes in foraging location have also been linked to lower forage quality in ungulates (Street et al. 2016; Mason et al. 2017; Alston et al. 2020), and in lizards, foraging opportunity costs associated with shade-seeking have been severe enough to cause population collapses under climate change (Sinervo et al. 2010). However, reductions in foraging rate associated with shade-seeking did not occur in sparrow-weavers in this study, suggesting that these patterns may be species- and context-specific.

Indeed, sparrow-weavers in shadier home ranges appeared to experience less severe trade-offs between foraging and thermoregulation than those in sunnier home ranges, using shade at lower *T*_air_ and experiencing lower average operative temperatures [sensu Bakken (1976); Robinson et al. (1976)], Operative temperatures at midday were 7–16 °C cooler in shaded compared to sunlit microsites at our study site (Whyte 2023). Lower operative temperatures in shaded microsites apparently delayed the onset of panting with increasing *T*_air_, such that sparrow-weavers in shadier home ranges were able to maintain higher peck rates than those in sunnier home ranges as temperatures increased. These conditions may also account for the similar overall peck rates of sparrow-weavers in shadier home ranges and conspecifics in sunnier home ranges, despite the former apparently foraging less at most T_air_ values during our study. Assuming peck rates are positively correlated with food intake rates (we could not verify this, as most food items were too small to be identified), shade availability may therefore be an important determinant of sparrow-weaver home range quality in terms of foraging opportunities as *T*_air_ increases with advancing climate change. Whether the same is true for other species and other habitats likely depends on the extent to which shade-seeking is associated with foraging opportunity costs.

Sparrow-weavers with access to surface water foraged more over the entire *T*_air_ range of this study, had higher overall peck rates and sought shade at higher *T*_air_ compared to conspecifics without water access. However, although they did drink water from the troughs during our observations, this was rare. Furthermore, we did not observe differences in panting behaviour between sparrow-weavers with access to water and those without. Regular drinking may allow other arid-zone bird species to replenish water losses associated with sustained evaporative heat dissipation and thereby continue foraging in sunlit microsites at high *T*_air_ (Bartholomew and Cade 1963; Davies 1982; Speakman and Król 2010; Czenze et al. 2020). For example, recent work on arid-zone larks revealed interspecific variation in shade-seeking behaviour correlated with water-dependence: regularly drinking species continued foraging in the sun at higher *T*_air_ compared to non-drinking species (Orolowitz et al. 2023). However, regularly drinking larks also commenced panting at lower *T*_air_ than non-drinkers, providing support for the proposed role of evaporative cooling in driving the observed patterns of behaviour (Orolowitz et al. 2023). The failure of sparrow-weavers with access to water to regularly drink, and the fact they did not pant more, or at cooler *T*_air,_ than those without water, mean it remains unclear whether the birds’ behaviour reflected among-individual variation in capacity for sustained evaporative cooling, although we cannot rule this possibility out.

Instead, we suggest another possibility: home ranges incorporating livestock watering troughs may have reduced availability of grass seed and insects (food for sparrow-weavers, Ferguson 1988), compared to those without, on account of piosphere effects (i.e. trampling by livestock, which removes vegetation and reduces biodiversity around water points; Andrews 1998; Egeru et al. 2015). Previous work at this site showed that water troughs also attract large numbers of granivorous birds (Abdu et al. 2018), likely increasing competition for seeds. Resource depletion associated with piospheres and increased competition could force sparrow-weavers to forage more to obtain sufficient food, as experimentally demonstrated in other arid-zone species (e.g. Tieleman and Williams 2002). We cannot rule out the possibility that T_air_ and hence operative temperature were slightly cooler in the immediate vicinity of water troughs because of evaporation, which is a potential explanation for why birds did not pant earlier in these home ranges, and why they sought shade later. However, this possibility seems unlikely, as birds in home ranges with water troughs foraged more than birds without water across all *T*_air._ This suggests that the need for greater foraging effort in these home ranges existed independently of any cooling effect of the water and was likely associated with resource depletion in the piospheres. The notion of reduced food availability in the vicinity of water sources could be tested directly by measuring seed availability in home ranges with and without water troughs, or potentially indirectly by quantifying sparrow-weaver body condition.

Group-living in animals is common in harsh environments globally (Rubenstein and Lovette 2007; Griesser et al. 2017; Lukas and Clutton-Brock 2017) and much research has focussed on the hypothesis that individuals in groups benefit through load-lightening (e.g. Rubenstein and Lovette 2007; Rubenstein 2011), via increased survival and reduced interannual variation in reproductive success. The load-lightening hypothesis has also led to predictions that animals in larger groups will maintain higher fitness than those in smaller groups as environments become hotter and drier under climate change (Bourne et al. 2020b). Reduced individual time allocation to vigilance behaviour could permit individuals in larger groups to forage more intensively, potentially reducing the time they spend foraging at high operative temperatures, thereby reducing heat exposure without compromising daily foraging intake. However, most studies explicitly testing interactions between temperature and group size on various proxies of fitness have not detected buffering effects of additional group members (Bourne et al. 2020b, 2023; Guindre-Parker and Rubenstein 2020; Van de Ven et al. 2020a, b; D’Amelio et al. 2022; Riehl and Smart 2022; however, see Capilla-Lasheras et al. 2021). Our data reveal interactions between *T*_air_ and group size but, like previous studies, do not clearly support the hypothesis that load-lightening buffers negative effects of heat.

In conclusion, our findings emphasise the potential for home range thermal heterogeneity to buffer birds against periods of hot weather, particularly when trade-offs between shade-seeking and foraging are weak. The reductions in panting we observed in sparrow-weavers occupying shadier home ranges allowed birds to maintain foraging intensity while reducing time spent foraging with increasing *T*_air_. This suggests increasing shade availability through effective conservation of trees such as camelthorns (*V*. erioloba) may assist in thermally buffering birds as climate change advances. In addition to reducing the severity of fitness costs arising from behavioural trade-offs, shady vegetation is also becoming increasingly important as a thermal refuge for savanna birds during extreme heat events (Strydom et al. 2025). However, reductions in opportunity costs associated with shady vegetation may be more constrained in some species, as evident from studies of larks*,* southern fiscals and southern yellow-billed hornbills (Cunningham et al. 2015; van de Ven et al. 2019; Orolowitz et al. 2023), among others. Simplistic conservation interventions like tree planting may also have unintended negative consequences in savanna environments (Bond et al. 2019): management of shade in these environments therefore needs to be approached in an ecologically sensitive manner. Future research should seek to better understand inter- and intraspecific variation in the opportunity costs associated with shade-seeking in order to identify species likely to benefit from conservation actions to maintain or increase shade availability and to identify how this can be done responsibly.

## Data Availability

The datasets used and/or analysed during the current study are available from the corresponding author on reasonable request.
